# Association between late age-related macular degeneration and dietary intake of copper, iron, zinc and selenium: a 2005–2008 NHANES cross-sectional observational study

**DOI:** 10.1186/s12886-025-04156-y

**Published:** 2025-05-30

**Authors:** Yicheng Lu, Chen Li, Yueqi Liu, Tianhong Wu, Peirong Lu

**Affiliations:** Department of Ophthalmology, The First Affiliated Hospital of Soochow University, 899 Pinghai Road, Suzhou, Jiangsu 215006 China

**Keywords:** Age-related macular degeneration, Diet, Copper, NHANES

## Abstract

**Background:**

Age-related macular degeneration (AMD) is a significant global cause of visual impairment. Our study seeks to explore the relationship between the intake of copper, iron, zinc, selenium in diet and late AMD.

**Method:**

In this cross-sectional study, we utilized data from the National Health and Nutrition Examination Survey (NHANES) conducted during 2005–2008. We employed three logistic regression models with or without adjustments to examine the association between dietary copper, iron, zinc, selenium and late AMD.

**Result:**

Our study involved 4996 individuals aged 40 years and above with graded fundus pictures and dietary trace element intake data from a representative sample. The levels of copper intake were linked to a reduced risk of late AMD, resulting in odds ratios (OR) of 0.24 (95% confidence interval [CI] = 0.13–0.46), 0.38 (95% CI = 0.16–0.90), and 0.37 (95% CI = 0.17–0.82) for crude model 1, adjusted model 2, and adjusted model 3 respectively. The intake levels of dietary iron, zinc and selenium showed an inverse correlation with the prevalence of late AMD in the crude model; ORs (95% CI) were as follows: Iron − 0.92 (0.86, 0.97); Zinc– 0.88 (0.81, 0.96); Selenium– 0.98 (0.97, 0.99). However, in model 2 and 3, no significant association was observed between these three elements and late AMD. In subgroup analysis divided by age, there was only a significant inverse correlation observed between late AMD and copper intake in 70–85 years of age group.

**Conclusion:**

Our findings suggest a higher dietary copper intake may be associated with a reduced risk of late AMD, with the protective effect remaining significant among individuals aged 70–85 years. While no significant association was identified between dietary intake of iron, zinc, selenium and AMD after adjusting for confounding factors. Further research is warranted to elucidate the mechanism underlying the relationship.

**Supplementary Information:**

The online version contains supplementary material available at 10.1186/s12886-025-04156-y.

## Introduction

Age-related macular degeneration (AMD), driven by global population aging, stands as a prominent cause of blindness on a global scale. It affected approximately 20 million people in the US as of 2019 [1]. The number of AMD patients is expected to rise to approximately 288 million worldwide by 2040 [2]. The early-stage AMD is characterized by the accumulation of extracellular deposits, known as drusen, at the interface between the retinal pigment epithelium (RPE) and Bruch’s membrane, as well as the neurosensory retina. Progression from early to moderate AMD is marked by increasing drusen size and the emergence of pigmentary changes in the retina. In late stage of AMD, geographic atrophy (GA) and /or abnormal blood vessels growth in the macular region may occur [3]. Currently, intravitreal injections of anti-vascular endothelial growth factor (VEGF) medications are clinically administered for the treatment of neovascularization in late AMD. However, there are no clinically viable therapies for GA that can either slow disease progression or restore lost vision. Therefore, there is a need to find preventive methods for treating late AMD.

AMD has been associated with various risk factors examined across diverse studies such as age, gender differences, social habits like smoking and alcohol consumption, and environmental influences [4–7]. Currently, dietary patterns have emerged as potential influencers on AMD development [8]. The Mediterranean diet, inherently abundant in antioxidants, is shown to mitigate the likelihood of developing AMD [8, 9]. Two prospective studies have identified high-glycemic diets containing high levels of carbohydrate food or edible sugar, as a contributing factor in the advancement of AMD [10, 11]. A dietary analysis conducted among participants from both Age-Relate Eye Disease Study 1 (AREDS1) and AREDS2 trials indicated that increased intake of minerals, vitamins, and carotenoids was correlated with diminished risks of late-stage AMD particularly geographic atrophy [12].

Copper is an essential trace element found abundantly in seafood, animal viscera, nuts and beans within the human diet. It plays a critical role in various biological processes such as aerobic respiration, antioxidant defense and extracellular matrix biosynthesis [13, 14]. There are reports suggesting its involvement in chronic ailments including diabetes and cardiovascular diseases [15]. Furthermore, in ocular physiology, copper acts as a co-factor for multiple ocular enzymes like copper-zinc superoxide dismutase (Cu-Zn SOD), which forms part of the primary antioxidant system [16, 17]. The activation of Cu-Zn SOD may be facilitated through its interaction with antioxidant1 (Atox1) [17].

The pathogenesis of AMD has been linked to oxidative stress and reduced antioxidant capacity [18]. Copper levels in the RPE and choroid complex of AMD patients have been found to be significantly lower than those of controls, impacting the management of oxidative stress [16]. Additionally, iron [19], zinc [20] and selenium [21] are essential trace elements that regulate antioxidant functions vital for ocular health, with zinc playing a particularly crucial role. Zinc exhibits antioxidative property through various mechanisms including inhibiting reduced nicotinamide adenine dinucleotide phosphate (NADPH) oxidase, acting as a cofactor for SOD and so on [22, 23]. In terms of specific components, the AREDS1 trial revealed that individuals utilizing zinc supplements (80 mg zinc oxide), alongside with antioxidants like vitamin C, vitamin E, and beta-carotene exhibited reduced progression rates towards advanced stages of AMD over six years when compared to control groups [24].

Nevertheless, the definitive establishment of the relationship between dietary intake of copper, iron, zinc, and selenium and AMD remains elusive. Currently, only an analysis for AREDS1 and AREDS2 cohorts using validated food frequency questionnaires indicated that dietary intake of copper, iron and selenium may slow down the progression of late AMD (HR (hazard ratio = 0.7–0.9; *p* < 0.05) [12]. Therefore, our study aims to investigate the association between intake levels of these four trace elements (copper, iron, zinc and selenium) and AMD.

## Method

### Sample and population

The Centers for Disease Control and Prevention (CDC) conducts an annual comprehensive nationwide population study known as the National Health and Nutrition Examination Survey (NHANES). This survey aims to assess the overall health status of the US population. NHANES is a cross-sectional series of interviews and exams conducted among the civilian, non-institutionalized population of the United States. It comprises a comprehensive interview questionnaire that collects information on diverse health issues, medications, and dietary habits, as well as a physical examination. NHANES published data covering the period from 2005 to 2008, which included calculations of nutrient intake based on thorough interviews regarding the participants’ dietary information. Additionally, it provided data from graded fundus pictures of individuals aged 40 and older. This extensive, nationally representative dataset enables researchers to investigate the association between copper, iron, zinc and selenium consumption from self-reported dietary information and AMD diagnosis based on fundus photography. However, this study still had a relatively small sample size for late AMD (*n* = 49).

Since the examination and evaluation for AMD were conducted exclusively during the 2005–2006 and 2007–2008 cycles, and considering the availability of trace element intake data (copper, iron, zinc and selenium) for these periods, we opted to utilize the publicly accessible NHANES data from 2005 to 2008 to investigate the potential association between AMD and dietary trace element intake. Our analysis focused on individuals aged 40 and above, who constitute the target demographic for AMD screenings. This study does not fall under the category of research involving human subjects as it utilized pre-existing de-identified public data. Therefore, ethical approval and consent were not necessary. All NHANES participants had previously provided informed consent, and the procedures were approved by the National Center for Health Statistics Research Ethics Review Board. NHANES employs a stratified, multistage sampling methodology to provide an accurate estimate of illness prevalence in the US population, necessitating the use of a weighting mechanism. Our statistical analyses were performed using this weighted data set, with only participants possessing complete information on both AMD and dietary trace intake (copper, iron, zinc and selenium) being included in our study.

### Variable

The study’s independent variables included copper, iron, zinc, and selenium intakes as determined through dietary interviews conducted during the 2005–2008 cycles. According to Recommended Dietary Allowance (RDA), the proper value for daily dietary intake of copper, iron, zinc and selenium is 900 mcg, 8 mg, 11 mg and 55 mcg for adult men. Given that the majority of women in our sample were elderly, we chose the standard of RDA for post-menopause women. Specifically, the RDAs for copper, iron, zinc, and selenium were set at 900 mcg, 8 mg, 8 mg, and 55 mcg for women, respectively [25]. Intake levels of copper (mg/day), iron (mg/day), zinc (mg/day), and selenium (mcg/day) were assessed using two 24-hour food recalls. The first recall interview was conducted in person, while the second was collected via a phone call 3 to 10 days later. In this study, we utilized the mean of these two 24-hour food recalls. Trained interviewers administered the dietary recall using the automated multiple-pass method developed by the United States Department of Agriculture (USDA) [26]. To convert ingested food and beverages into nutrient intakes, NHANES used the USDA’s Food and Nutrient Database for Dietary Studies (FNDDS), which relies on the National Nutrient Database for Standard Reference (NNDSR) of the USDA to access information on food composition [27]. The FNDDS and NNDSR databases associated with each NHANES survey cycle differ. To calculate the copper, iron, zinc, and selenium content in foods consumed during the 2005–2008 cycle, NHANES used the trace element addendum to the FNDDS3 food composition database that supplied necessary data for accurate input and calculations. Further information is available on the website: https://wwwn.cdc.gov/Nchs/Nhanes/2005-2006/DR1TOT_D.htm, https://wwwn.cdc.gov/Nchs/Nhanes/2007-2008/DR1TOT_E.htm, https://wwwn.cdc.gov/Nchs/Nhanes/2005-2006/DR2TOT_D.htm and https://wwwn.cdc.gov/Nchs/Nhanes/2007-2008/DR1TOT_D.htm.

The primary outcome of our study was the presence of AMD, as determined by NHANES using fundus photography. Forty-five-degree non-mydriatic digital images of the retina were captured from survey participants aged 40 years and older using the Canon Non-Mydriatic Retinal Camera CR6-45NM with a Canon 10D camera back (6.3 megapixels per image). Technicians conducting the exams received training in the use of the digital imaging system. The room was darkened to facilitate natural pupil dilation during image capture, without pharmacologic dilation. Digital images were evaluated by graders at the University of Wisconsin using a modified version of the Wisconsin Age-Related Maculopathy Grading Classification System. Following systematic grading, if there was disagreement between the first two graders regarding diagnosis, a third grader evaluated the eye. If two out of three graders disagreed, an adjudicator reviewed the image to make a final decision. Early AMD is defined by drusen larger than 500 μm within a grid area and/or pigmentary abnormalities; late AMD is characterized by signs of exudative age-related macular degeneration and/or geographic atrophy. When retinal pictures were available for both eyes, analysis focused on the eye with more severe findings.

Potential covariates included age (years), gender (male, female), race/ethnicity (non-Hispanic white, non-Hispanic black, Mexican American/other Hispanic/other race), marriage status (never married, widowed/divorced/separated, living with partner/married), PIR (a ratio of family income to poverty threshold, classified into < 2.5, 2.5-5.0, > 5.0), educational attainment (less than 11th grade, High School Grad/Some College or AA [Associate of Arts] degree, College Graduate or above), body mass index (BMI) (in kg/m^2^), smoke status (never, former, now), hypertension diagnosis (no, yes) diabetes diagnosis (no, yes, board line) and total energy intake. These covariates were collected through either a questionnaire or medical examination by trained examiners from the CDC. Further details about this information can be found at the following website: https://wwwn.cdc.gov/Nchs/Nhanes/.

### Statistical analysis

In accordance with NHANES guidelines, all analyses utilized sample weights derived from stratified, multistage, probabilistic sampling designs. We incorporated sampling weights, strata, and sampling units in our statistical analysis to accommodate the complex sampling design. Continuous variables were described using the mean ± standard deviation (SD). Categorical variables were presented as unweighted frequencies (n) and weighted percentages (%). Weighted one-way ANOVA tests and Chi-square tests were used to compare differences in confounders between the no AMD and early AMD group, late AMD, any AMD groups as appropriate. Weighted logistic regression models were used to investigate whether levels of trace element intake are independently associated with AMD, accounting for various potential confounding factors. Model 1 did not contain any covariates; Model 2 included demographic adjustments for age, sex, race, marriage, education, poverty, and total energy intake; Model 3 extended Model 2 by including physical additional adjustments for BMI, smoking status, hypertension, and diabetes. Missing data occurred among the covariates. Due to limitations of various imputation methods in accurately reflecting the true situation, we chose to exclude missing data from our analysis. R (version 4.3.2) was used throughout the entire data analysis process.

## Results

**Fig. 1:**
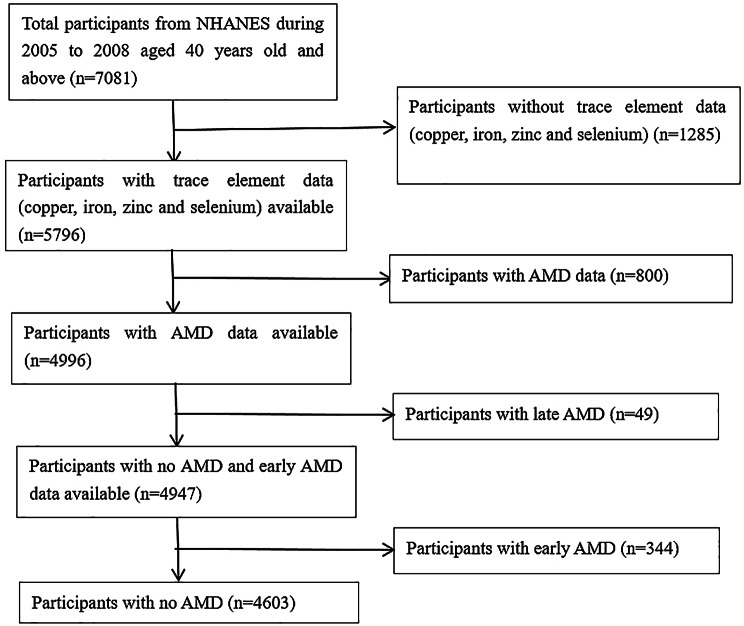
Flowchart of sample selection

A total of 7081 individuals aged 40 years and older participated in the NHANES study from 2005 to 2008. Among them, 1285 individuals were excluded due to insufficient trace element data (copper, iron, zinc and selenium). Subsequently, an additional 800 participants without AMD data were excluded. Among the remaining participants, 344 were diagnosed with early AMD, 49 with late AMD, and 4603 showed no signs of AMD. (as shown in Fig. 1).

The data in Table 1 presented the demographic information, BMI, health-related activities, total energy intake, and trace element intake of the individuals. A significant difference was identified in age, BMI, total energy intake, PIR (poverty income index), marriage, race, education, smoking, and hypertension among the three groups under weighted one-way ANOVA analysis (*p* < 0.05). The results were especially significant in age (55.85 vs. 67.46 vs. 77.08), total energy intake (2033.58 vs. 1858.06 vs. 1553.67), marriage, and race (*p* < 0.001). There was no significant difference in sex and diabetes between these 3 groups. Copper, iron, zinc and selenium all demonstrated significant difference and similar trend between these three groups (*p* < 0.001), with no AMD as the highest and late AMD as the lowest. We observed that only the late AMD group for men exhibited a zinc intake below the RDA standard of 11 mg/day (10.80 mg/day) (supplementary Table 1).

**Table 1:** Characteristics of included people based on AMD status in NHANES 2005–2008

Characteristic, unweighted *n* (weighted percentage) / mean (SD)	No AMD (*n* = 4603)	Early AMD (*n* = 344)	Late AMD (*n* = 49)	*P* value
**Age, years**	55.85 (11.18)	67.48 (12.07)	77.08 (7.36)	< 0.001
**BMI,kg/m** ^**2**^	29.12 (6.36)	29.05 (6.32)	26.38 (4.33)	0.006
**Total energy intake, kcl**	2033.58 (783.59)	1858.06 (654.69)	1553.67 (448.94)	< 0.001
**Copper, mg/day**	1.38 (0.85)	1.33 (1.05)	1.02 (0.33)	< 0.001
**Iron, mg/day**	15.59 (7.61)	15.23 (6.95)	12.20 (5.21)	0.001
**Zinc, mg/day**	12.31 (9.07)	11.68 (9.14)	9.04 (4.01)	< 0.001
**Selenium, mcg/day**	107.97 (48.28)	95.34 (39.20)	78.05 (33.46)	< 0.001
**Sex**				0.21
Male	2267 (45.7)	177 (45.4)	18 (29.5)	
Female	2336 (54.3)	167 (54.6)	31 (70.5)	
**PIR**				0.001
< 2.5	2129 (34.2)	180 (43.1)	27 (58.3)	
2.5-5.0	1194 (29.7)	87 (30.8)	10 (23.9)	
> 5.0	976 (30.8)	44 (16.8)	8 (13.9)	
Unknown	304 (5.3)	33 (9.3)	4 (3.9)	
**Marriage**				< 0.001
Married/Living with partner	3022 (69.2)	195 (61.3)	18 (31.0)	
Widowed/Divorced/Separated	1251 (24.0)	141 (37.5)	29 (66.4)	
Never married	328 (6.8)	8 (1.2)	2 (2.6)	
Unknown	2 (0.0)	0 (0.0)	0 (0.0)	
**Race**				< 0.001
Mexican American/Other Hispanic/Other race	1159 (12.7)	69 (9.0)	2 (1.5)	
Non-Hispanic white	2490 (77.4)	245 (87.6)	44 (93.9)	
Non-Hispanic black	954 (9.9)	30 (3.4)	3 (4.6)	
**Education**				0.040
Less than11th Grade	1286 (16.5)	105 (22.1)	10 (19.3)	
High School Grad/Some College or AA degree	2339 (54.5)	178 (54.6)	32 (69.4)	
College Graduate or above	977 (29.0)	61 (23.2)	7 (11.3)	
Unknown	1 (0.0)	0 (0.0)	0 (0.0)	
**Smoking status**				0.001
Never	2189 (49.0)	149 (39.5)	22 (37.7)	
Former	1490 (30.3)	143 (43.2)	21 (48.0)	
Now	923 (20.7)	52 (17.3)	6 (14.3)	
Unknown	1 (0.0)	0 (0.0)	0 (0.0)	
**Hypertension**				0.014
No	2112 (59.1)	150 (46.0)	21 (46.7)	
Yes	2484 (40.8)	194 (54.0)	28 (53.3)	
Unknown	7 (0.1)	0 (0.0)	0 (0.0)	
**Diabetes**				0.84
No	3822 (87.7)	280 (85.9)	41 (82.4)	
Yes	682 (10.2)	52 (11.6)	7 (15.3)	
Board line	94 (2.0)	12 (2.4)	1 (2.3)	
Unknown	5 (0.0)	0 (0.0)	0 (0.0)	

Unweighted n, all other analyses are weighted. AMD, age-related macular degeneration; BMI, body mass index; PIR, poverty income ratio

As shown in, in Model 1, all four elements were associated with a reduced risk of late AMD without any adjustment (all *p* < 0.01). However, after adjusting demographic information in model 2, only copper intake was found to decrease the risk of late AMD (OR = 0.38, 95%CI = 0.16–0.90, *p* = 0.030). Even in Model 3 adjusted for demographic and physical information, copper intake remained significantly linked to a lower risk of late AMD (OR = 0.37, 95%CI = 0.17–0.82, *p* = 0.018), while no significant association was observed between late AMD and the other three elements. Significant correlation between trace element and early or any AMD was only observed in selenium intake in crude Model 1. (*p* < 0.001) (Table 2). However, no significant difference was found under adjustment in Model 2 and Model 3. The detailed number of cases were listed below Table 2.

In order to decrease the influence of the age gap between AMD and no AMD group, a subgroup analysis divided by age was shown in Table 3. A significant negative association was observed between copper intake and late AMD (model 1: 0.48 (0.25,0.89), *p* = 0.022; model 2: 0.46 (0.24,0.89), *p* = 0.023; model 3: 0.46 (0.21,0.99), *p* = 0.047) in 70–85 age group. Because of the insufficient sample size of late AMD in 55–69 age group and 40–54 age group (both *n* < 5), the weighted logistic model on late AMD in these 2 subgroups is not available. No significant associations were observed between copper intake and early AMD or any AMD after stratifying on age.

**Table 2:** Weighted logistic model of late, early and any AMD and copper, iron, zinc

	Model	Late AMD OR (95%CI)	*p*-value	Early AMD OR (95%CI)	*p*-value	Any AMD OR (95%CI)	*p*-value
Copper intake	Model 1	0.24 (0.13, 0.46)	< 0.001	0.91 (0.66, 1.27)	0.58	0.83 (0.56, 1.22)	0.33
	Model 2	0.38 (0.16, 0.90)	0.030	1.02 (0.81, 1.27)	0.87	0.98 (0.76, 1.25)	0.84
	Model 3	0.37 (0.17, 0.82)	0.018	1.03 (0.85, 1.25)	0.72	0.98 (0.77, 1.26)	0.88
Iron intake	Model 1	0.92 (0.86, 0.97)	0.006	0.99 (0.97, 1.01)	0.52	0.99 (0.97, 1.00)	0.11
	Model 2	0.94 (0.89, 1.00)	0.062	1.00 (0.97, 1.02)	0.90	0.99 (0.97, 1.02)	0.56
	Model 3	0.95 (0.90,1.02)	0.14	1.00 (0.98, 1.04)	0.84	0.99 (0.97, 1.02)	0.60
Zinc intake	Model 1	0.88 (0.81, 0.96)	0.005	0.99 (0.96, 1.02)	0.51	0.98 (0.94, 1.02)	0.30
	Model 2	0.94 (0.85, 1.04)	0.21	1.00 (0.98, 1.02)	0.96	1.00 (0.98, 1.02)	0.85
	Model 3	0.97 (0.87, 1.08)	0.54	1.00 (0.98, 1.02)	0.90	1.00 (0.98, 1.02)	0.77
Selenium intake	Model 1	0.98 (0.97, 0.99)	< 0.001	0.99 (0.99, 1.00)	0.001	0.99 (0.99, 1.00)	< 0.001
	Model 2	0.99 (0.98, 1.00)	0.16	1.00 (0.99, 1.02)	0.24	1.00 (0.99, 1.00)	0.14
	Model 3	0.99 (0.98, 1.01)	0.41	1.00 (0.99, 1.01)	0.14	1.00 (0.99, 1.00)	0.065

Model 1:4603 no AMD cases, 344 early AMD cases, 49 late AMD cases, adjusted for none

Model 2: 4600 no AMD cases with 344 early AMD cases, 49 late AMD cases, adjusted for age, sex, race, marriage and education

Model 3: 4557 no AMD cases with 343 early AMD cases, 48 late AMD cases, model 2 +total energy intake, BMI, hypertension, diabetes, smoking status

**Table 3:** Weighted subgroup logistic model of late, early and any AMD and copper divided by age

Ages, year	Late AMD			Early AMD			Any AMD		
	Model 1	Model 2	Model 3	Model 1	Model 2	Model 3	Model 1	Model 2	Model 3
40–54	NA	NA	NA	0.87 (0.55, 1.36) *p* = 0.52	0.98 (0.67, 1.43) *p* = 0.89	1.01 (0.72, 1.40) *p* = 0.97	0.86 (0.55, 1.35) *p* = 0.50	0.98 (0.69, 1.40) *p* = 0.91	0.91 (0.35, 2.36) *p* = 0.83
55–69	NA	NA	NA	1.00 (0.50, 2.00) *p* = 0.90	0.93 (0.38, 2.24) *p* = 0.86	0.99 (0.49, 1.98) *p* = 0.97	0.95 (0.44, 2.09) *p* = 0.90	0.88 (0.33, 2.36) *p* = 0.80	0.93 (0.39, 2.21) *p* = 0.87
70–85	0.48 (0.25, 0.89) *p* = 0.022	0.46 (0.24, 0.89) *p* = 0.023	0.46 (0.21, 0.99) *p* = 0.047	1.13 (0.87, 1.46) *p* = 0.34	1.08 (0.83, 1.39) *p* = 0.56	1.10 (0.89, 1.37) *p* = 0.34	1.06 (0.85, 1.33) *p* = 0.58	1.10 (0.84, 1.44) *p* = 0.48	1.10 (0.89, 1.37) *p* = 0.34

Model 1 (adjusted for none): 40-54 years old: 1958 no AMD cases with 41 early AMD cases and 1 late AMD case

55-69 years old: 1646 no AMD cases with 98 early AMD cases and 4 late AMD cases

70-85 years old: 999 no AMD cases with 205 early AMD cases and 44 late AMD cases

Model 2 (adjusted for age, sex, race, marriage and education): 40-54 years old: 1956 no AMD cases with 41 early AMD cases and 1 late AMD case

55-69 years old:1645 no AMD cases with 98 early AMD cases and 4 late AMD cases

70-85 years old: 999 no AMD cases with 205 early AMD cases and 44 late AMD cases

Model 3 (model 2 +total energy intake, BMI, hypertension, diabetes, smoking status): 40-54 years old: 1945 no AMD cases with 41 early AMD cases and 1 late AMD case

55-69 years old: 1628 no AMD cases with 98 early AMD cases and 4 late AMD cases

70-85 years old: 984 no AMD cases with 204 early AMD cases and 43 late AMD cases

## Discussion

In this research, we conducted an analysis to investigate the potential association between the dietary intake of four trace elements and AMD in individuals aged 40 and above, utilizing data from two cycles of NHANES (2005–2006 and 2007–2008). Our findings indicate that higher copper consumption is associated with a reduced risk of late-stage AMD among individuals aged 40 and older in the US, even after accounting for potential confounding variables and meeting the standard of RDA. In subgroup analyses, the inverse association between copper intake and late AMD remained significant among those aged 70–85 years after adjusting for confounding variables (model 2: 0.46 (0.24, 0.89), *p* = 0.023; model 3: 0.46 (0.21,0.99), *p* = 0.047). This finding is consistent with the results of a cohort study for AREDS1 and AREDS2 [12]. In contrast, no significant associations were observed between selenium or iron intake and late AMD, which does not align with the findings of the AREDS 1 and AREDS 2 study [12].

Oxidative stress is a key factor in the pathogenesis of AMD. It is widely acknowledged that retina requires a high demand of oxygen, even exceeding that of the brain when adjusted for weight. Due to its unique anatomical and metabolic characteristics, the retina provides an optimal environment for the generation of reactive oxygen species (ROS). These features include high oxygen consumption by the outer retina-RPE complex, substantial cumulative irradiation, abundant photosensitizers in the neurosensory retina and RPE, and the retina becomes even more vulnerable to oxidative damage due to its polyunsaturated fatty acid-rich photoreceptor outer segment membranes [28]. When ROS production exceeds antioxidant capacity, ROS-mediated oxidative stress can lead to advanced AMD characterized by neurodegeneration, particularly photoreceptor cell death, resulting in irreversible vision loss [29]. High levels of ROS can damage mitochondrial DNA, triggering cell death by permeabilizing the mitochondrial outer membrane and releasing of specific proteins into the cytoplasm due to mitochondrial injury [30]. Specifically, research has demonstrated a positive correlation between mitochondrial injury, reduced mass and number of mitochondria, and the severity of AMD [31]. Consequently, unable to meet the high energy demands of photoreceptors, the RPE may ultimately undergo apoptosis [32]. Trace elements have been involved as regulators of oxidative stress in the aged retina. Iron accumulation might lead to reduced efficiency of intracellular antioxidative defense systems, causing the aging retina to undergo oxidative stress-induced cell death [19]. Zinc possesses antioxidant properties by inhibiting reduced NAPDH oxidase [33]. The evidence for the role of selenium in the retina is limited, but animal studies have demonstrated that it can be involved in the antioxidant mechanisms that prevent oxidant damage by activating glutathione peroxidase [34].

Over the years, it has been demonstrated that copper also plays a role in modulating oxidative stress. Copper deficiency can cause cardiac damage by promoting the production of ROS and reducing the activity of antioxidant enzymes in the heart compared to other tissues [35]. Elevated serum cholesterol and triglycerides have also been associated with decreased copper levels [36]. During pregnancy, a lack of copper may lead to embryonic death, structural abnormalities, as well as persistent biochemical, neurological, and immunological irregularities [37]. Copper deficiency seems to affect certain diseases characterized by oxidative stress. Animal and cell culture models have illustrated that copper deficiency can result in an increase in ROS and oxidative damage to lipids, DNA, and proteins. Studies on copper-deficient rat models have revealed a reduced activity of Cu-Zn SOD and ceruloplasmin, along with elevated superoxide anions, which indirectly lowers glutathione peroxidase (GPX) activity and may contribute to lipid peroxidation and DNA oxidative damage [38, 39]. A significant portion of the mitochondrial copper pool is utilized by cytochrome c oxidase, and the activity of this critical cuproenzyme of mitochondria may be decreased due to copper deficiency, leading to an enhanced production of ROS [40, 41].

Given the crucial role of oxidative stress in the pathogenesis of AMD, copper intake may impact AMD by influencing oxidative stress. Reduced levels of copper have been found in the RPE and choroid complex of individuals with AMD [16]. Intracellular copper depletion triggers apoptosis of RPE and retinal cells [42]. Homocysteine (Hcy) and homocysteine-thiolactone (HcyTL) are toxic as they can lead to abnormal chelation of the trace element such as copper, thereby altering the structure and function of proteins [43, 44]. Interestingly, in AMD patients, an increase in Hcy and HcyTL was negatively correlated with plasma copper level, potentially reducing Cu-Zn SOD amounts, leading to increased oxidative stress and subsequent damage to RPE cells [45]. A report from the AREDS 1 and AREDS 2 study demonstrated similar results to ours, indicating that copper intake was linked to reduced late-stage AMD risk [12]. Our findings, combined with evidence from oral supplementation and laboratory investigation, suggest that consuming copper is associated with a decreased risk of developing late AMD.

However, in adjusted logistic models, we did not find any significant association between iron, zinc, selenium, and AMD. Dietary nutrient intake from the AREDS 1 and AREDS 2 studies indicated that higher intake of iron, magnesium and selenium was linked to decreased late AMD risk; however, no association was found between dietary zinc intake and late AMD [12]. Conversely, the Select Eye Endpoints (SEE) study revealed that dietary selenium intake showed no association with AMD development [46]. Additionally, a randomized placebo-controlled clinical trial on high-dose supplementation with vitamins C and E, beta carotene, and zinc for AMD from AREDS suggested that an antioxidant plus zinc supplement should be considered for preventing late AMD [24]. The population-based Rotterdam study demonstrated that a high dietary intake of nutrients with antioxidant properties including zinc can reduce risk of early AMD [47]. The inconsistent result may be attributed to different factors included in the adjustment of multinomial logistics regression as well as differences in dosage between daily intake and additional supplements [24].

It is important to acknowledge that our study has limitations with respect to the potential presence of unknown confounding factors including the combinatory effect among different trace elements when consumed together and the inability to establish a causal relationship between copper consumption levels and late AMD due to its cross-sectional nature. Besides, considering the relatively small sample size of late AMD, we were unable to use residual method to adjust total energy intake in trace elements, but we did include total energy intake in our logistic model to reduce confounding effect. Last, further investigation is required to clarify the mechanism that underlies the association between copper intake and the development of late AMD.

## Conclusion

The findings of this study suggest that increased copper intake is associated with a reduced prevalence of late AMD in the US. While no significant association was identified between dietary intake of iron, zinc, selenium and AMD after adjusting for confounding factors. In order to clarify the mechanism between copper intake and the development of late AMD, further investigation into the mechanism linking copper intake and the development of late AMD is warranted.

## Electronic supplementary material

Below is the link to the electronic supplementary material.

Supplementary Material 1

## Data Availability

The datasets generated and analysed during the current study are available in online repositories. The names of the repository/repositories and accession number(s) can be found at: NHANES 2005–2006. https://wwwn.cdc.gov/nchs/nhanes/continuousnhanes/default.aspx?BeginYear=2005.NHANES 2007–2008.https://wwwn.cdc.gov/nchs/nhanes/continuousnhanes/default.aspx?BeginYear=2007.

## Abbreviations

NHANES: National Health and Nutrition Examination Survey
OR: Odds Ratio
HR: Hazard Ratio
CI: Confidence Interval
SD: Standard Deviation
RPE: Retinal Pigment Epithelium
GA: Geographic Atrophy
VEGF: Vascular Endothelial Growth Factor
Cu-Zn: Copper-Zinc
SOD: Superoxide Dismutase
Atox1: Antioxidant1
NADPH: Nicotinamide Adenine Dinucleotide Phosphate
AREDS: Age-related Eye Disease Study
CDC: the Centers for Disease Control and Prevention
USDA: the United States Department of Agriculture
FNDDS: Food and Nutrient Database for Dietary Studies
PIR: Poverty Income Ratio
BMI: Body Mass Index
Hcy: Homocysteine
HcyTL: Homocysteine-Thiolactone

## References

[1] Rein DB, Wittenborn JS, Burke-Conte Z, Gulia R, Robalik T, Ehrlich JR, Lundeen EA, Flaxman AD. Prevalence of Age-Related macular degeneration in the US in 2019. JAMA Ophthalmol. 2022;140(12):1202–8. 10.1001/jamaophthalmol.2022.4401. 36326752 10.1001/jamaophthalmol.2022.4401 PMC9634594

[2] Wong WL, Su X, Li X, Cheung CM, Klein R, Cheng CY, Wong TY. Global prevalence of age-related macular degeneration and disease burden projection for 2020 and 2040: a systematic review and meta-analysis. Lancet Glob Health. 2014;2(2):e106–116. 10.1016/S2214-109X(13)70145-1. 25104651 10.1016/S2214-109X(13)70145-1

[3] Fleckenstein M, Schmitz-Valckenberg S, Chakravarthy U. Age-Related macular degeneration: A review. JAMA. 2024;331(2):147–57. 10.1001/jama.2023.26074. 38193957 10.1001/jama.2023.26074 PMC12935482

[4] Heesterbeek TJ, Lorés-Motta L, Hoyng CB, Lechanteur YTE, den Hollander AI. Risk factors for progression of age-related macular degeneration. Ophthalmic Physiol Opt. 2020;40(2):140–70. 10.1111/opo.12675. 32100327 10.1111/opo.12675 PMC7155063

[5] Joachim N, Mitchell P, Rochtchina E, Tan AG, Wang JJ. Incidence and progression of reticular Drusen in age-related macular degeneration: findings from an older Australian cohort. Ophthalmology. 2014;121(4):917–25. 10.1016/j.ophtha.2013.10.043. 24332537 10.1016/j.ophtha.2013.10.043

[6] Sakurada Y, Sugiyama A, Kikushima W, Yoneyama S, Tanabe N, Matsubara M, Iijima H. Pseudodrusen pattern and development of late age-related macular degeneration in the fellow eye of the unilateral case. Jpn J Ophthalmol. 2019;63(5):374–81. 10.1007/s10384-019-00680-9. 31267312 10.1007/s10384-019-00680-9

[7] Clemons TE, Milton RC, Klein R, Seddon JM, Ferris FL. 3rd. Risk factors for the incidence of advanced Age-Related macular degeneration in the Age-Related eye disease study (AREDS) AREDS report 19. Ophthalmology. 2005;112(4):533–9. 10.1016/j.ophtha.2004.10.047. 15808240 10.1016/j.ophtha.2004.10.047 PMC1513667

[8] Pameijer EM, Heus P, Damen JAA, Spijker R, Hooft L, Ringens PJ, Imhof SM, van Leeuwen R. What did we learn in 35 years of research on nutrition and supplements for age-related macular degeneration: a systematic review. Acta Ophthalmol. 2022;100(8):E1541–52. 10.1111/aos.15191. 35695158 10.1111/aos.15191 PMC9796889

[9] Merle BM, Silver RE, Rosner B, Seddon JM. Adherence to a mediterranean diet, genetic susceptibility, and progression to advanced macular degeneration: a prospective cohort study. Am J Clin Nutr. 2015;102(5):1196–206. 10.3945/ajcn.115.111047. 26490493 10.3945/ajcn.115.111047 PMC4625588

[10] Chiu CJ, Milton RC, Klein R, Gensler G, Taylor A. Dietary carbohydrate and the progression of age-related macular degeneration: a prospective study from the Age-Related eye disease study. Am J Clin Nutr. 2007;86(4):1210–8. 10.1093/ajcn/86.4.1210. 17921404 10.1093/ajcn/86.4.1210

[11] Kaushik S, Wang JJ, Flood V, Tan JS, Barclay AW, Wong TY, Brand-Miller J, Mitchell P. Dietary glycemic index and the risk of age-related macular degeneration. Am J Clin Nutr. 2008;88(4):1104–10. 10.1093/ajcn/88.4.1104. 18842800 10.1093/ajcn/88.4.1104

[12] Agrón E, Mares J, Clemons TE, Swaroop A, Chew EY, Keenan TDL. Dietary nutrient intake and progression to late Age-Related macular degeneration in the Age-Related eye disease studies 1 and 2. Ophthalmology. 2021;128(3):425–42. 10.1016/j.ophtha.2020.08.018. 32858063 10.1016/j.ophtha.2020.08.018 PMC7902480

[13] Huffman DL, O’Halloran TV. Function, structure, and mechanism of intracellular copper trafficking proteins. Annu Rev Biochem. 2001;70:677–701. 10.1146/annurev.biochem.70.1.677. 11395420 10.1146/annurev.biochem.70.1.677

[14] Puig S, Thiele DJ. Molecular mechanisms of copper uptake and distribution. Curr Opin Chem Biol. 2002;6(2):171–80. 10.1016/s1367-5931(02)00298-3. 12039001 10.1016/s1367-5931(02)00298-3

[15] Tiffany-Castiglioni E, Hong S, Qian Y. Copper handling by astrocytes: insights into neurodegenerative diseases. Int J Dev Neurosci. 2011;29(8):811–8. 10.1016/j.ijdevneu.2011.09.004. 21968186 10.1016/j.ijdevneu.2011.09.004

[16] Erie JC, Good JA, Butz JA, Pulido JS. Reduced zinc and copper in the retinal pigment epithelium and choroid in age-related macular degeneration. Am J Ophthalmol. 2009;147(2):276–e282271. 10.1016/j.ajo.2008.08.014. 18848316 10.1016/j.ajo.2008.08.014

[17] Yang D, Xiao P, Qiu B, Yu HF, Teng CB. Copper chaperone antioxidant 1: multiple roles and a potential therapeutic target. J Mol Med (Berl). 2023;101(5):527–42. 10.1007/s00109-023-02311-w. 37017692 10.1007/s00109-023-02311-w

[18] Decanini A, Nordgaard CL, Feng X, Ferrington DA, Olsen TW. Changes in select redox proteins of the retinal pigment epithelium in age-related macular degeneration. Am J Ophthalmol. 2007;143(4):607–15. 10.1016/j.ajo.2006.12.006. 17280640 10.1016/j.ajo.2006.12.006 PMC2365890

[19] Zhao T, Guo X, Sun Y. Iron accumulation and lipid peroxidation in the aging retina: implication of ferroptosis in Age-Related macular degeneration. Aging Dis. 2021;12(2):529–51. 10.14336/ad.2020.0912. 33815881 10.14336/AD.2020.0912 PMC7990372

[20] Grahn BH, Paterson PG, Gottschall-Pass KT, Zhang Z. Zinc and the eye. J Am Coll Nutr. 2001;20(2 Suppl):106–18. 10.1080/07315724.2001.10719022. 11349933 10.1080/07315724.2001.10719022

[21] Al-Bassam L, Shearman GC, Brocchini S, Alany RG, Williams GR. The potential of Selenium-Based therapies for ocular oxidative stress. Pharmaceutics. 2024;16(5). 10.3390/pharmaceutics16050631. 10.3390/pharmaceutics16050631 PMC11125443 38794293

[22] Prasad AS. Zinc: an antioxidant and anti-inflammatory agent: role of zinc in degenerative disorders of aging. J Trace Elem Med Biol. 2014;28(4):364–doi371. 10.1016/j.jtemb.2014.07.019. 25200490 10.1016/j.jtemb.2014.07.019

[23] Jarosz M, Olbert M, Wyszogrodzka G, Mlyniec K, Librowski T. Antioxidant and anti-inflammatory effects of zinc. Zinc-dependent NF-kappaB signaling. Inflammopharmacology. 2017;25(1):11–24. 10.1007/s10787-017-0309-4. 28083748 10.1007/s10787-017-0309-4 PMC5306179

[24] A randomized, placebo-controlled, clinical trial of high-dose supplementation with vitamins C and E, beta carotene, and zinc for age-related macular degeneration and vision loss: AREDS report no. 8. Arch Ophthalmol. 2001; 119(10):1417–1436;10.1001/archopht.119.10.1417 10.1001/archopht.119.10.1417 PMC1462955 11594942

[25] Institute of Medicine Panel on Micronutrients. Dietary reference intakes for. In: Vitamin A, Vitamin K, Arsenic, editors. Boron, chromium, copper, iodine, iron, manganese, molybdenum, nickel, silicon, vanadium, and zinc. Washington (DC): National Academies Press (US); 2001. 25057538

[26] National center for health Statistics national health and nutrition examination survey MEC in-person dietary interviewers procedures manual. Centers for Disease Control and Prevention. 2006. https://wwwn.cdc.gov/nchs/data/nhanes/2005-2006/manuals/DIETARY_MEC.pdf

[27] Agricultural research service what we eat in America food categories 2009–2010. U.S. Department of Agriculture. 2012. https://www.ars.usda.gov/ARSUserFiles/80400530/pdf/0910/wweia_2009_2010_data.pdf

[28] Jabbehdari S, Handa JT. Oxidative stress as a therapeutic target for the prevention and treatment of early age-related macular degeneration. Surv Ophthalmol. 2021;66(3):423–doi440. 10.1016/j.survophthal.2020.09.002. 32961209 10.1016/j.survophthal.2020.09.002

[29] Curcio CA. Photoreceptor topography in ageing and age-related maculopathy. Eye (Lond). 2001;15(Pt 3):376–83. 10.1038/eye.2001.140. 11450761 10.1038/eye.2001.140

[30] Westphal D, Kluck RM, Dewson G. Building blocks of the apoptotic pore: how Bax and bak are activated and oligomerize during apoptosis. Cell Death Differ. 2014;21(2):196–205. 10.1038/cdd.2013.139. 24162660 10.1038/cdd.2013.139 PMC3890949

[31] Fisher CR, Ferrington DA. Perspective on AMD pathobiology: A bioenergetic crisis in the RPE. Invest Ophthalmol Vis Sci. 2018;59(4):Amd41–7. 10.1167/iovs.18-24289. 30025108 10.1167/iovs.18-24289 PMC5989860

[32] He Y, Tombran-Tink J. Mitochondrial decay and impairment of antioxidant defenses in aging RPE cells. Adv Exp Med Biol. 2010;664:165–83. 10.1007/978-1-4419-1399-9_20. 20238015 10.1007/978-1-4419-1399-9_20

[33] Blasiak J, Pawlowska E, Chojnacki J, Szczepanska J, Chojnacki C, Kaarniranta K. Zinc and autophagy in Age-Related macular degeneration. Int J Mol Sci. 2020;21(14). 10.3390/ijms21144994. 10.3390/ijms21144994 PMC7404247 32679798

[34] Akil M, Bicer M, Menevse E, Baltaci AK, Mogulkoc R. Selenium supplementation prevents lipid peroxidation caused by arduous exercise in rat brain tissue. Bratisl Lek Listy. 2011;112(6):314–7. 21692404

[35] Liu Y, Miao J. An emerging role of defective copper metabolism in heart disease. Nutrients. 2022;14(3). 10.3390/nu14030700. 10.3390/nu14030700 PMC8838622 35277059

[36] Saari JT. Copper deficiency and cardiovascular disease: role of peroxidation, glycation, and nitration. Can J Physiol Pharmacol. 2000;78(10):848–55. 10.1139/cjpp-78-10-848. 11077985 10.1139/cjpp-78-10-848

[37] Keen CL, Clegg MS, Hanna LA, Lanoue L, Rogers JM, Daston GP, Oteiza P, Uriu-Adams JY. The plausibility of micronutrient deficiencies being a significant contributing factor to the occurrence of pregnancy complications. J Nutr. 2003; 133(5 Suppl 2):1597s-1605s;10.1093/jn/133.5.1597S 10.1093/jn/133.5.1597S 12730474

[38] Song M, Schuschke DA, Zhou Z, Chen T, Pierce WM Jr., Wang R, Johnson WT, McClain CJ. High Fructose feeding induces copper deficiency in Sprague-Dawley rats: a novel mechanism for obesity related fatty liver. J Hepatol. 2012;56(2):433–40. 10.1016/j.jhep.2011.05.030. 21781943 10.1016/j.jhep.2011.05.030 PMC3261305

[39] Hawk SN, Lanoue L, Keen CL, Kwik-Uribe CL, Rucker RB, Uriu-Adams JY. Copper-deficient rat embryos are characterized by low superoxide dismutase activity and elevated superoxide anions. Biol Reprod. 2003;68(3):896–903. 10.1095/biolreprod.102.009167. 12604640 10.1095/biolreprod.102.009167

[40] Dodani SC, Leary SC, Cobine PA, Winge DR, Chang CJ. A targetable fluorescent sensor reveals that copper-deficient SCO1 and SCO2 patient cells prioritize mitochondrial copper homeostasis. J Am Chem Soc. 2011;133(22):8606–16. 10.1021/ja2004158. 21563821 10.1021/ja2004158 PMC3106114

[41] Medlock AE, Hixon JC, Bhuiyan T, Cobine PA. Prime real estate: metals, cofactors and MICOS. Front Cell Dev Biol. 2022;10:892325. 10.3389/fcell.2022.892325. 35669513 10.3389/fcell.2022.892325 PMC9163361

[42] Hyun HJ, Sohn JH, Ha DW, Ahn YH, Koh JY, Yoon YH. Depletion of intracellular zinc and copper with TPEN results in apoptosis of cultured human retinal pigment epithelial cells. Investig Ophthalmol Vis Sci. 2001;42(2):460–5. 11157883

[43] Hughes WM, Rodriguez WE, Rosenberger D, Chen J, Sen U, Tyagi N, Moshal KS, Vacek T, Kang YJ, Tyagi SC. Role of copper and homocysteine in pressure overload heart failure. Cardiovasc Toxicol. 2008;8(3):137–44. 10.1007/s12012-008-9021-3. 18679830 10.1007/s12012-008-9021-3 PMC2671677

[44] Jakubowski H. Pathophysiological consequences of homocysteine excess. J Nutr. 2006;136(6 Suppl). 1741s-1749s;. 10.1093/jn/136.6.1741S 16702349

[45] Bharathselvi M, Biswas S, Raman R, Selvi R, Coral K, Narayanansamy A, Ramakrishnan S, Sulochana KN. Homocysteine & its metabolite homocysteine-thiolactone & deficiency of copper in patients with age related macular degeneration - A pilot study. Indian J Med Res. 2016;143(6):756–62. 10.4103/0971-5916.192026. 27748300 10.4103/0971-5916.192026 PMC5094115

[46] Christen WG, Darke AK, Gaziano JM, Glynn RJ, Goodman PJ, Minasian LM, Thompson IM Jr. Age-related macular degeneration in a randomized trial of selenium and vitamin E in men: the select eye endpoints (SEE) study (SWOG S0000B). Acta Ophthalmol. 2021;99(2):e285–7. 10.1111/aos.14538. 32701201 10.1111/aos.14538

[47] Ho L, van Leeuwen R, Witteman JC, van Duijn CM, Uitterlinden AG, Hofman A, de Jong PT, Vingerling JR, Klaver CC. Reducing the genetic risk of age-related macular degeneration with dietary antioxidants, zinc, and ω-3 fatty acids: the Rotterdam study. Arch Ophthalmol. 2011;129(6):758–66. 10.1001/archophthalmol.2011.141. 21670343 10.1001/archophthalmol.2011.141

