# Supplementary material for: Association between late age-related macular degeneration and dietary intake of copper, iron, zinc and selenium: a 2005–2008 NHANES cross-sectional observational study
Source: BMC Ophthalmol. 2025 May 30;25:327. doi: 10.1186/s12886-025-04156-y (PMC12125803; doi:10.1186/s12886-025-04156-y)
Supplement: Supplementary file 1 — Supplementary Material 1 [file 12886_2025_4156_MOESM1_ESM.docx]

| Mean/SD | No AMD | | Early AMD | | Late AMD | |
| --- | --- | --- | --- | --- | --- | --- |
|  | Male | Female | Male | Female | Male | Female |
| Copper, mg/day | 1.57 (1.03) | 1.23 (0.61) | 1.42 (1.08) | 1.25 (1.01) | 1.15 (0.30) | 0.97 (0.33) |
| Iron, mg/day | 17.97 (8.03) | 13.58 (6.32) | 17.38 (7.82) | 13.45 (5.56) | 15.32 (6.02) | 10.90 (4.26) |
| Zinc, mg/day | 14.67 (11.52) | 10.33 (5.59) | 13.13 (7.18) | 10.48 (10.36) | **10.80 (4.64)** | 8.30 (3.52) |
| Selenium, mcg/day | 126.52 (51.57) | 92.32 (38.98) | 107.55 (40.35) | 85.19 (35.23) | 88.55 (36.12) | 73.67 (31.74) |

Supplementary Table 1: Copper, iron, zinc and selenium intake levels stratified by AMD subgroup and sex (value below the standard of RDA are highlighted in bold)
